# 异基因造血干细胞移植后供者型白血病复发转换类型1例

**DOI:** 10.3760/cma.j.cn121090-20250625-00298

**Published:** 2025-11

**Authors:** 瑞靖 李, 敏 熊, 根 王, 珍 崔, 君利 朱

**Affiliations:** 河北燕达陆道培医院，廊坊 065201 Hebei Yanda Lu Daopei Hospital, Langfang 065201, China

患者，女，13岁，2016年9月（患者4岁余）以“齿龈出血”起病。查血常规：WBC 10.32×10^9^/L、HGB 77 g/L、PLT 24×10^9^/L；胸骨骨髓形态：增生明显活跃，原始、幼稚淋巴细胞占94.5％，考虑为急性淋巴细胞白血病（ALL）-L2。流式细胞术：R2区细胞约占62.5％，表达CD10、HLA-DR、CD19、CD5、cyCD79a，考虑带有T系标记的common B-ALL。染色体核型：46, XX, t（1;12）（q21;p13）[3]/45,idem,−3,add（9）（p13）x2, −18, +mar[1]/46, XX[12]。白血病融合基因筛查阴性；白血病基因分型：IKZF1（全基因缺失型）阳性；双色荧光杂交染色：ETV6/RUNX1及MLL均阴性；根据骨髓MICM诊断B-ALL。予中危B-ALL-08年方案化疗，期间间断复查骨髓持续处于完全缓解状态。2019年5月进入维持治疗阶段。2019年8月19日患者白血病复发。骨髓形态：增生Ⅱ级，原始、幼稚淋巴细胞占有核细胞87.4％；流式细胞术：58.45％细胞（占有核细胞）表达CD19、CD38、CD81、cCD79a、HLA-DR、CD24、cBcl-2、CD22，部分表达CD10，不表达CD34、CD20、TDT、CD13、CD33、CD117、CD15、CD11b、kappa、lambda、cIgM、MPO、cCD3、CD7、CD14、CD64、CD11c，为恶性幼稚B淋巴细胞；染色体核型：46,XX[3]；白血病融合基因筛查阴性；22种ALL基因突变筛查：TP53 R282P、EZH2 G159W突变阳性；先天免疫缺陷基因筛查（免疫失调）：阴性；予VLP（长春地辛3 mg每周1次+地塞米松7 mg每12 h 1次×8 d+左旋门冬酰胺酶10 000 IU/d×4 d）方案化疗。后予氟达拉滨（40 mg/d，第1～3天）+去甲氧柔红霉素（10 mg/d，d 2）+环磷酰胺（0.3 g/d，第1～3天）方案化疗。患者形态学缓解，微小残留病（MRD）阳性，后予输注CD19-嵌合抗原受体T细胞（CAR-T细胞）。后复查骨髓为完全缓解状态。拟行父供女半相合异基因造血干细胞移植，予阿糖胞苷（Ara-C）+全身放疗+环磷酰胺/抗人胸腺细胞免疫球蛋白（ATG）/司莫司汀预处理方案，11月12日输注供者骨髓造血干细胞，13日输注供者外周血造血干细胞，14日输注第三方脐带血造血干细胞。+16 d白细胞植活，+19 d血小板植活。此后多次复查骨髓穿刺，原发病缓解状态，嵌合率完全供者状态。为预防复发，予小剂量地西他滨（10 mg，第1～7天）化疗3次。

移植后3年9个月余（2023年7月18日）患者血小板开始进行性下降，复查骨髓象：有核细胞增生极度活跃，原始粒细胞增高占39.5％；单核细胞增高，可见原始及幼稚单核细胞占8.0％，考虑急性髓系白血病（AML）；染色体核型：46,XY,t（9;11）（p21.3;q23.3）[29]；骨髓嵌合率为完全供者状态。外周血嵌合率（CD3^+^细胞）为完全供者状态。MLL∷AF9（KMT2A∷MLLT3）融合基因定量分析：30.694％。予高三尖杉酯碱（3 mg/d，第1～3天）+地西他滨（10 mg/d，第1～3天）+Ara-C（25 mg，每12 h 1次，第1～3天）方案化疗。2023年8月1日外周血细胞形态见原始细胞占6％，后予以柔红霉素+维奈克拉加强化疗。8月28日复查骨髓象，骨髓形态缓解，特殊MRD检测（流式细胞法）：异常表型髓系原始细胞占有核细胞0.23％；染色体核型：46,XY[3]。拟行异基因造血干细胞移植（供者为其母亲，HLA高分辨配型7/10位点相合，A+型供O+型），预处理方案为Ara-C+白消安+Flu/ATG/司莫司汀。9月20日起连续3 d分别输注供者骨髓、供者外周血及第三方脐带血造血干细胞。10月4日（+12 d）白细胞、血小板植活。移植后多次复查原发病未见异常，嵌合率为完全供者状态。目前患者移植后1年6个月。

讨论：本例患者移植后骨髓MICM确诊复发，且染色体为供者来源，嵌合率为完全供者状态，说明患者骨髓细胞来源于供者，复发的肿瘤细胞来自供者细胞。患者多次化疗，长期应用免疫抑制剂，治疗过程导致骨髓造血环境受损，引起供者干细胞出现突变诱发白血病。也不除外供者存在某些癌症基因，在患者体内可出现癌变可能。供者细胞在患者体内患白血病，再次输注供者造血干细胞及供者淋巴细胞治疗无效，积极创造移植条件，更换供者再次行异基因造血干细胞移植。患者目前已移植术后1年6个月，治疗效果好。此病例为异基因造血干细胞移植后供者型复发提供了治疗经验。

